# Editorial: New opportunities in drug design for the management and treatment of type 2 diabetes

**DOI:** 10.3389/fphar.2023.1187057

**Published:** 2023-04-06

**Authors:** Hassan Rasouli, Teodorico C. Ramalho, Jelena B. Popović-Djordjević, Hari Prasad Devkota

**Affiliations:** ^1^ Medical Biology Research Center (MBRC), Kermanshah University of Medical Sciences, Kermanshah, Iran; ^2^ Department of Chemistry, Federal University of Larvas, Larvas, Brazil; ^3^ Department of Food Technology and Biochemistry, Faculty of Agriculture, University of Belgrade, Belgrade, Serbia; ^4^ Graduate School of Pharmaceutical Sciences, Kumamoto University, Kumamoto, Japan

**Keywords:** drug design, diabetes mellitus, type 2 diabetes, inhibitor, hypoglycemic agents

Today, diabetes mellitus (DM), a chronic and complex metabolic disease, remains a major concern for countries worldwide. According to statistics from the World Health Organization (WHO), approximately 422 million people worldwide suffer from DM, and this number is expected to increase by 2050 (WHO, 2022). Various DM subtypes have been identified to date, and specific clinical therapies have been developed to ameliorate the health complications associated with each subtype. The growing body of evidence suggests that type 2 DM (T2DM) or non-insulin dependent DM accounts for nearly 90% of all diabetic cases, while type 1 DM (T1DM) or insulin-dependent DM accounts for only 5% of cases (Rasouli et al., 2020). Currently, a range of small molecule inhibitors, peptides, and other therapies are available in global pharmaceutical markets to inhibit or modulate the dysfunction of enzymatic pathways involved in the pathogenesis of T2DM (Dahlén et al., 2022; Popovic-Djordjevic et al., 2018; , 2022; Bailey et al., 2023). Despite significant advancements in understanding various dimensions of DM onset, unfortunately, there is still no ultimate drug or therapeutic strategy to completely prohibit the progress of DM (Popovic-Djordjevic et al., 2018). Recent high throughput transcriptomics, genomics, and metabolomics studies have shed light on the molecular, cellular, and physiological facets of DM, providing scientists with a better understanding of how to manage this disease (Gan et al., 2020).

**FIGURE 1:**
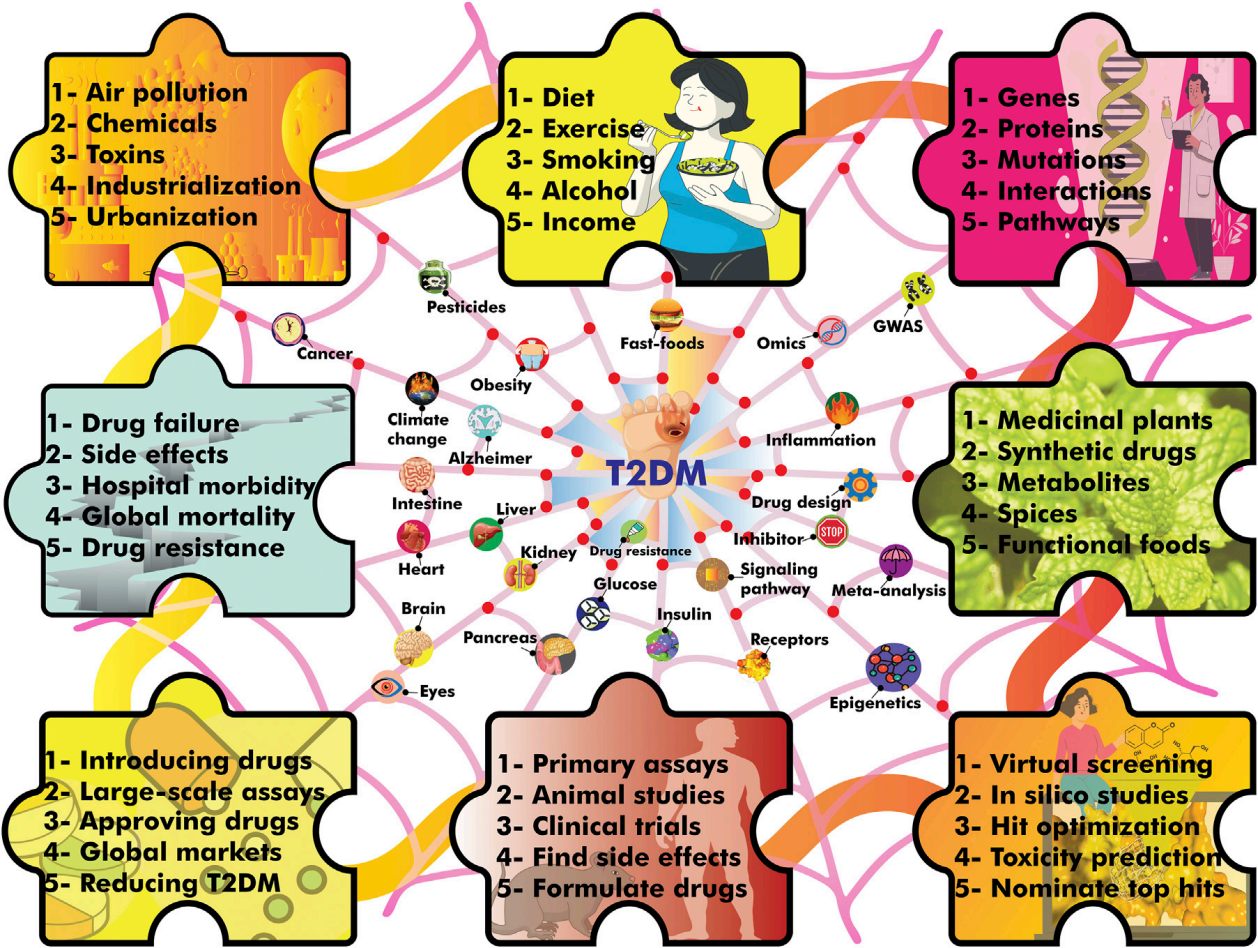
A concise depiction of the intricate interplay between environmental and genetic risk factors, lifestyle, efforts for drug design, drug failure and mortality of T2DM.

Research indicates that T2DM is linked to numerous chronic conditions (Popovic-Djordjevic et al., 2018), with various environmental and genetic risk factors contributing to the development of this metabolic disease (Figure 1). Most hypoglycemic drugs have been designed to address T2DM. Notably, α-glucosidase and α-amylase inhibitors, which are targeted to inhibit carbohydrate metabolizing enzymes, have been among the most thoroughly researched hypoglycemic drugs. While a plethora of natural and synthetic substances have been examined for their ability to mitigate the dysregulation of specific enzymes involved in T2DM pathogenesis, only a few have been approved for clinical application (Rasouli et al., 2020; Jugran et al., 2021). The available evidence suggests that some of these approved drugs may elicit side effects, exacerbating the health complications of diabetic individuals (Dahlén et al., 2022). Moreover, the mass production of synthetic hypoglycemic drugs necessitates specialized facilities, ample financial resources, and skilled technicians.

To surmount these formidable obstacles, researchers have scoured natural reservoirs to identify and characterize new hypoglycemic agents with less adverse effects (Jugran et al., 2021). Notably, the deleterious health consequences of T2DM for affected individuals have fueled a marked upswing in studies centered on this research domain. Furthermore, the advent of cutting-edge omics-based techniques has revealed an abundance of information about T2DM’s molecular elements, including signaling pathways, enzymes, genes, transcription factors, and other key factors. Additionally, the rapid strides made in artificial intelligence, machine learning, deep learning, and other computational approaches have empowered scientists to devise sophisticated methods for early T2DM diagnosis and establish robust healthcare management strategies to enhance T2DM control.

This Research Topic aims to elucidate new opportunities for the treatment of T2DM and provides a platform to highlight novel studies conducted in this field. In this Research Topic, Kim et al. conducted a study on a novel small molecule ligand known as (*E*)-2-methoxy-4-(3-(4-methoxyphenyl) prop-1-en-1-yl) phenol (MMPP), which has been shown to enhance adipogenesis and glucose uptake by binding to the PPARγ receptor. This ligand has previously exhibited significant biological activities and is now being repurposed for the treatment of T2DM. In this study, the authors used an integrated approach that combined experimental assays with computational docking studies to elucidate the binding mode of MMPP to the PPARγ ligand-binding site. Their findings reveal that MMPP can significantly reduce the side effects of thiazolidinediones, which are other agonists of the PPARγ receptor. By improving glucose uptake and modulating adipogenesis-related factors, MMPP holds promise as a promising therapeutic option for the management of T2DM.

The manuscript by Zhuang et al. reports a study to investigate the potential hypoglycemic and anti-obesity properties of *Dendrobium* mixture, a patented Chinese botanical medicine. While prior research indicated that this medicinal herb possesses notable anti-inflammatory effects and improves glycolipid metabolism, the mechanisms of its active ingredients and potential mode of action remained uncertain. To elucidate the hypoglycemic potential of this botanical mixture, the authors employed an integrated experimental approach involving TMT-based quantitative proteomics, LC-MS/MS analysis, and advanced bioinformatics methods. Their findings suggest that the studied plant may improve insulin resistance, activate PPARγ, and significantly lower blood glucose, thus targeting several genetic factors implicated in T2DM and non-alcoholic fatty liver disease. Overall, this study provides novel insights into the therapeutic potential of *Dendrobium* mixture and highlights the importance of rigorous scientific investigation in identifying effective treatments for metabolic disorders.

Another study by Song et al. performed a systematic review and meta-analysis to assess the efficacy and safety of Chinese herbal medicine (CHM) in treating painful diabetic neuropathy. Their results suggest that CHM, when employed as a single treatment or as an adjunct therapy, exerts promising effects and is deemed safe for patients suffering from painful diabetic neuropathy. The authors’ rigorous analysis of high-quality clinical studies supports the notion that CHM constitutes a valuable substance for alleviating the complications of DM. Consequently, their findings endorse the adoption of CHM as a complementary treatment for managing painful diabetic neuropathy, and advocate for further research to validate its therapeutic potential in modern clinical studies. Finally, Fatoki et al. optimized the production of α-amylase inhibitor in solid culture using the *Streptomyces xinghaiensis* AAI-2 strain. Based on these results, the optimal pH and moisture content for producing α-amylase inhibitor using *S. xinghaiensis* were determined in this study. The authors utilized an interesting experimental methodology to produce effective α-amylase inhibitors.

In conclusion, this Research Topic has resulted in the publication of four papers that were co-authored by 29 scientists. The manuscripts included in this research Research Topic significantly demonstrate that numerous dimensions of T2DM remain to be discovered and understood, in order to enhance the therapeutic options currently available. Therefore, it is imperative to develop cost-effective strategies for producing novel hypoglycemic agents that minimize side effects while maximizing efficacy, as well as comprehending the molecular aspects of T2DM progression. These requirements are essential for further studies to reduce the number of people affected by diabetes and decrease the worldwide mortality rate associated with T2DM.
